# Quantitative correlation of rock fragment gradation parameters and TBM disc cutter efficiency: Insights from DEM simulations

**DOI:** 10.1371/journal.pone.0345490

**Published:** 2026-06-26

**Authors:** Jiawei An, Qianlong Wang, Jianxing Liao, Chaolin Wang, Yachen Xie, Yiqiang Lu, Fei He, Dabin Zhang

**Affiliations:** 1 College of Civil Engineering, Guizhou University, Guiyang, China; 2 State Key Laboratory of Intelligent Construction and Healthy Operation and Maintenance of Deep Underground Engineering, College of Water Resources and Hydropower, Sichuan University, Chengdu, China; 3 China Railway Eng. Equipment Group Co., Ltd., Zhengzhou, China; 4 School of Mechanical Engineering, Guizhou University, Guiyang, China; Henan Polytechnic University, CHINA

## Abstract

This study explores the relationship between rock fragment size distribution and disc cutter breaking efficiency of a tunnel boring machine (TBM) using discrete element simulations of indentation tests. Fragmentation behavior was analyzed under varying joint dip angles, joint spacings, and confining pressures, and cutter efficiency was evaluated by correlating fragment gradation parameters with specific energy (SE). The results show that cracks preferentially propagate along the weak joint interfaces, demonstrating that joint geometry controls stress redistribution and fracture evolution. With increasing joint dip angle, crack density and crushed zone extent first increase and then decrease. Reduced joint spacing enhances crack interaction and coalescence, resulting in lower energy demand. In contrast, increasing confining pressure suppresses tensile crack development, restricts crack opening displacement, and promotes shear-dominated failure, leading to finer fragments and higher SE. Strong linear correlations (R² > 0.80) between gradation parameters and SE indicate that fragment size distribution quantitatively reflects variations in energy dissipation during cutter and rock interaction.

## Introduction

Tunnel boring machines (TBMs) are widely used in the construction of highways, railways, and water conveyance tunnels [1–5], and have recently been applied to roadway excavation in coal mines [6]. During excavation, complex geological conditions, including jointed rock masses and heterogeneous lithologies, are frequently encountered and influence rock-breaking efficiency [7]. Cutter performance is governed by the mechanical interaction between disc cutters and the surrounding rock mass, and its reliable prediction is necessary for controlling construction cost and schedule [8]. However, direct monitoring of energy transfer and fracture processes remains difficult due to the confined and inaccessible tunnel face [9]. Rock fragments, as the primary products of TBM excavation, preserve information on in-situ rock conditions and fragmentation behavior [10]. Establishing a quantitative relationship between fragment characteristics and cutter efficiency is therefore important for performance evaluation in complex geological environments.

Previous studies have examined TBM rock-breaking efficiency through fragment analysis. Shaterpour-Mamaghani and Bilgin [11] showed that mechanically generated fragments reflect geological characteristics and that cutting efficiency can be evaluated using fragment size distribution. Heydari et al. [12] reported correlations among fragment gradation, operating parameters, and rock properties based on laboratory cutting and sieving tests. Tuncdemir et al. [13] identified the coarseness index (CI) as an indicator of cutter efficiency, demonstrating that specific energy decreases with increasing CI. Kim et al. [14] further evaluated the sensitivity of CI and maximum fragment size to cutter spacing and penetration. Other studies related fragment size distributions to thrust and torque [15], quantified relationships between fragment characteristics and operational parameters using image analysis [16], and assessed cutter efficiency through combined sieving and morphological analysis [17]. Altindag [18] established exponential correlations among penetration, CI, and mean fragment size. These studies indicate that fragment characteristics can reflect cutter–rock interaction and provide indirect measures of cutter efficiency. However, most existing investigations are based on macroscopic experimental observations, which remain largely empirical and may limit reliability under complex geological conditions.

To overcome experimental limitations, numerical simulations have been widely adopted to investigate TBM rock-breaking processes in jointed and heterogeneous rock masses. Coupled FEM–DEM and discrete element approaches have been used to analyze the influence of confining pressure, joint geometry, and crack evolution on fragmentation behavior [19–23]. Previous results show that increasing confining pressure reduces fragment size and alters fracture patterns, while variations in joint dip angle and spacing significantly affect crack propagation and fragment distribution. These studies demonstrate the capability of numerical methods to capture microscopic fracture processes and quantify the effects of geological factors on cutter performance. Nevertheless, existing simulations primarily focus on crack evolution mechanisms and specific model configurations. General relationships linking energy efficiency and fragment size distribution have not yet been systematically established, and modeling uncertainty may affect result consistency [24, 25].

In karst-dominated coal mine environments, rock masses are characterized by extensive jointing, heterogeneous lithologies, and high strength [26]. Such conditions require continuous adjustment of TBM operating parameters in response to geological variations [6]. Improper parameter selection may reduce tunneling efficiency and accelerate cutter wear, leading to operational instability [27]. A quantitative framework linking fragment characteristics to cutter operational parameters is therefore required to support performance evaluation and parameter optimization.

This study aims to establish a quantitative relationship between rock fragment size distribution and disc cutter energy efficiency based on discrete element simulations. By analyzing the effects of joint geometry and confining pressure on crack evolution and fragmentation behavior, an energy-based framework is proposed to link fragment gradation parameters with specific energy. The remainder of the paper is organized as follows. The numerical methodology and evaluation indices are presented in the theoretical background. Model development and calibration are described in the model construction and calibration part. The effects of joint characteristics and confining pressure on cutter efficiency are analyzed in the results and discussion. The potential engineering applications are discussed in the application section.

## 2. Theoretical background

### 2.1. Theory of the numerical method

**Fig 1:**
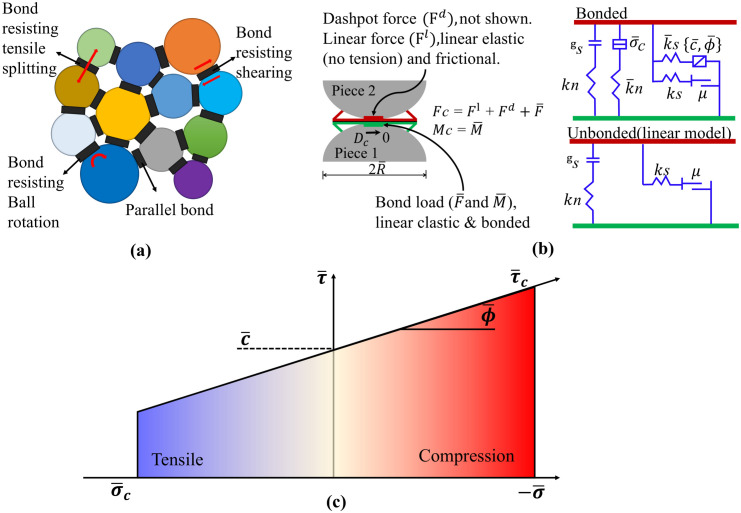
a. Particles bonded by LPBM; b. Behavior and rheological components of the LPBM; c. Strength envelope of the LPBM.

The discrete element method (DEM) is widely used in rock mechanics research, engineering damage analysis, and underground mining design, as it simulates macroscopic load-bearing behavior and deformation of rock and provides insight into crack evolution and failure processes [28–30]. Within DEM, the linear parallel bond model (LPBM) is commonly used to simulate rock fracture [31–33]. In the LPBM, particles are connected by bonded interfaces that resist forces and moments induced by particle rotation, tension, and shear (Fig 1a). In this study, commercial software particle flow code was employed to construct the LPBM [34]. For more detailed explanations of the LPBM, please refer to the PFC 6.0 manual.

Particle positions and contact states are updated during force transmission based on Newton’s second law [35]. The LPBM consists of two moment-resisting bonding interfaces and a linear-elastic contact interface (Fig 1b). The force–displacement law governs contact forces and moments, as shown in Eqs 1 and 2. When the interparticle force exceeds the bond strength, the bond breaks and the contact reverts to a linear model.


$$\begin{array}{c} \begin{array}{c} F_{c}=F^{\text{l}}+F^{d}+\bar{F} \end{array} \end{array}$$ (1)

$$\begin{array}{c} \begin{array}{c} M_{c}=\bar{M} \end{array} \end{array}$$ (2)

Where $F^{l}$is the linear force, $F^{d}$is the dashpot force, $\overset{―}{F}$ is the parallel-bond force, and $\bar{M}$is the parallel-bond moment. When the bond stress exceeds the tensile or shear strength defined by the Mohr–Coulomb criterion, bond failure occurs (Fig 1c). After bond breakage, the contact loses its moment-transmitting capacity and behaves as a frictional linear contact governed by normal and shear stiffness and the friction coefficient. The strength envelope is defined in Eq 3 [36].


$$\begin{array}{c} \begin{array}{c} {\overset{―}{\tau }}_{c}=\overset{―}{c}-\overset{―}{\sigma }tan\overset{―}{\phi } \end{array} \end{array}$$ (3)

where $\overset{―}{c}$ is the cohesion of the particle, $\overset{―}{\phi }$ the friction angle of the particles, and ${\overset{―}{\tau }}_{c}$ and $\overset{―}{\sigma }$ are the shear and normal stresses borne by the particles, respectively.

### 2.2. Indicators of fragments distribution

**Fig 2:**
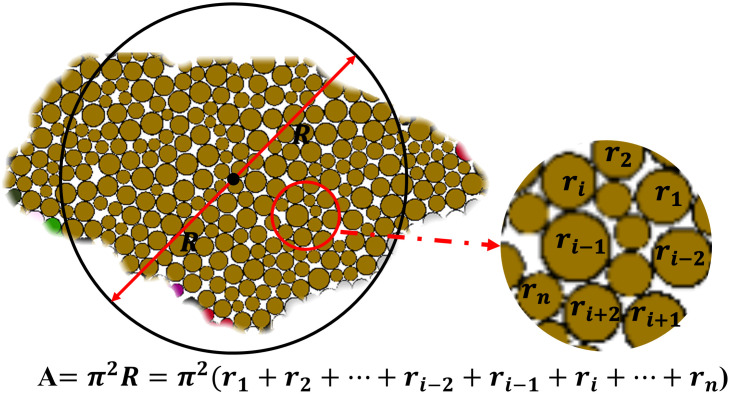
Illustration of the area equivalence principle.


To determine the particle size distribution of rock fragments, the broken particles are first grouped in the DEM numerical simulation. Specifically, particles in contact are classified into the same group, with each group considered an independent fragment unit. To facilitate quantitative comparison of particle sizes, each fragment is approximated as an equivalent circle. As shown in Fig 2, the principle involves calculating the total area A of the fragment and defining the equivalent circle diameter based on area conservation. The area equivalence principle is expressed by Eq 4.

$$\begin{array}{c} \begin{array}{c} A=\pi^{\text{2}}\left(r_{\text{1}}+r_{\text{2}}+\ldots +r_{i-\text{2}}+r_{i-\text{1}}+r_{i}+\ldots +r_{n}\right)=\sum_{i=\text{1}}^{n}\pi \overset{\text{2}}{\underset{i}{r}}=\pi R^{\text{2}} \end{array} \end{array}$$ (4)

where A denotes the equivalent circle area, R is the equivalent circle radius, and $r_{i}$ is the radius of the i particle in the fragment. This approach preserves fragment size characteristics and reduces the influence of irregular particle geometry.

**Fig 3:**
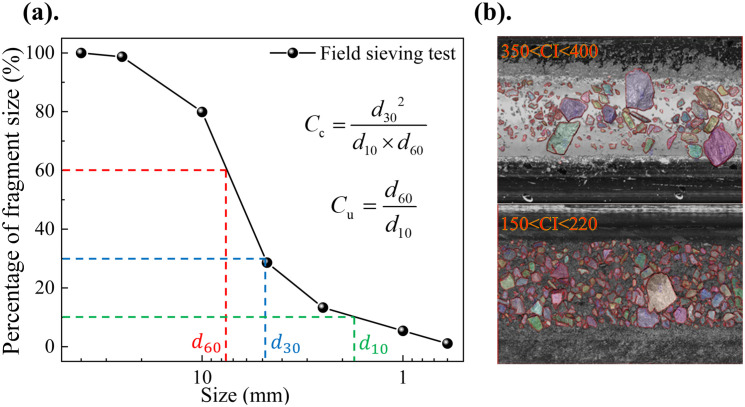
a. Illustration of fragment size distribution curve parameters; b. Illustration of two different coarseness index states.


A gradation curve was constructed based on the equivalent diameters of all rock fragments to quantify the fragment size distribution. The characteristic diameters $d_{\text{1}\text{0}}$, $d_{\text{3}\text{0}}$, and $d_{\text{6}\text{0}}$correspond to particle sizes at 10%, 30%, and 60% cumulative mass percentages, respectively (Fig 3a). Based on these values, the coefficient of uniformity $C_{u}$and the coefficient of curvature $C_{c}$were calculated through Eqs. 5 and 6.

$$\begin{array}{c} \begin{array}{c} C_{u}=\frac{d_{\text{6}\text{0}}}{d_{\text{1}\text{0}}} \end{array} \end{array}$$ (5)

$$\begin{array}{c} \begin{array}{c} C_{c}=\frac{\overset{\text{2}}{\underset{\text{3}\text{0}}{d}}}{d_{\text{1}\text{0}}d_{\text{6}\text{0}}} \end{array} \end{array}$$ (6)

where $C_{u}$ reflects the spread of particle sizes, with values closer to 1 indicating a more uniform distribution. $C_{c}$ describes the shape and continuity of the gradation curve. The definitions and interpretation of $C_{u}$ and $C_{c}$ are adopted from conventional geotechnical particle gradation criteria. In this study, these parameters are used as comparative descriptors of rock fragment size distribution rather than as direct soil classification indices.

In addition, the Coarseness Index (CI), proposed by Roxborough and Rispin [37], was used to quantify the overall coarseness of the fragments. CI is calculated from the mass percentage retained on each sieve:


$$\begin{array}{c} \begin{array}{c} x_{i}=\frac{w_{i}}{w_{sum}}\times \text{1}\text{0}\text{0}\% \end{array} \end{array}$$ (7)

$$\begin{array}{c} \begin{array}{c} CI=\sum_{i=\text{1}}^{n}x_{i} \end{array} \end{array}$$ (8)

where $w_{i}$is the mass of fragments retained on sieve i, $w_{sum}$ is the total fragment mass, and $x_{i}$ is the mass percentage retained on sieve i. A higher CI indicates coarser fragments and a lower degree of fragmentation, whereas a lower CI represents finer fragments and a higher degree of fragmentation (Fig 3b).

### 2.3. Indicators of cutter breaking efficiency

During indentation, the cutter-force response provides direct information on the mechanical interaction between the disc cutter and the rock mass. In the numerical model, the reaction forces acting on the cutters were continuously recorded throughout penetration. Their evolution reflects the main stages of rock fragmentation, including local compaction, crack initiation, crack propagation, crack coalescence, and fragment release. In general, force build-up indicates increasing resistance to penetration, whereas sudden fluctuations or local drops are associated with bond breakage and fragment detachment.

In the DEM simulations, the total mechanical work w was calculated by integrating the cutter reaction force over the penetration displacement. For the double-cutter indentation model, the total reaction force of the two cutters was used. Therefore, w can be expressed as:


$$\begin{array}{c} \begin{array}{c} w=\int_{\text{0}}^{u}F(u)\;du\approx \sum_{i=\text{1}}^{n}\frac{F_{i}+F_{i-\text{1}}}{\text{2}}\left({\text{u}}_{\text{i}}-{\text{u}}_{\text{i}-\text{1}}\right) \end{array} \end{array}$$ (9)

where F(u) is the total cutter reaction force and u is the penetration displacement.

Based on the calculated mechanical work, specific energy (SE) was adopted as a key indicator to evaluate TBM cutter breaking efficiency [38, 39]. SE represents the mechanical energy consumed per unit volume of fractured rock. Lower SE values correspond to lower energy consumption and thus higher cutting efficiency. SE is defined as:


$$\begin{array}{c} \begin{array}{c} SE=\frac{w}{A_{C}\cdot d} \end{array} \end{array}$$ (10)

where $A_{C}$ is the fractured rock area, w is the total mechanical work expended by the cutters, and d is the model thickness. In this study, the thickness of the two-dimensional numerical model was set to 1 mm.

## 3. Model construction and calibration

### 3.1. Engineering background

**Fig 4:**
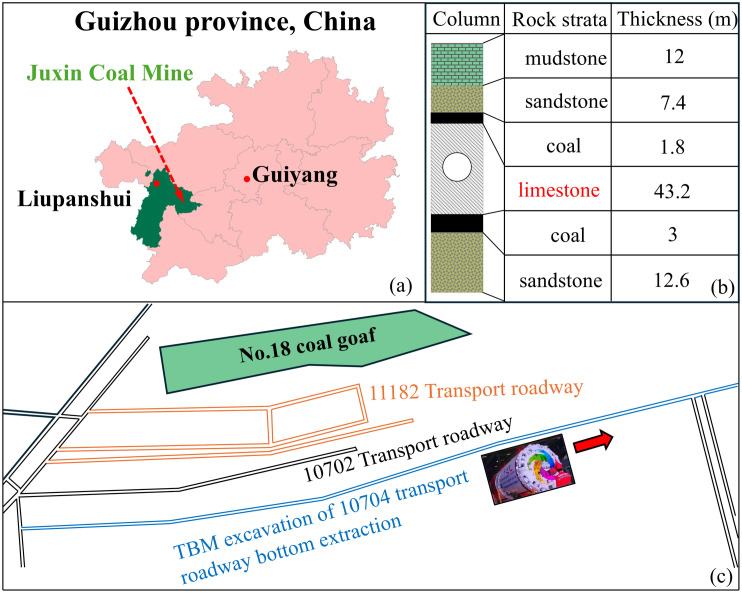
Geological features and TBM excavation layout of the #10704 transport roadway in Juxin Coal Mine. **(a)** Location of Juxin Coal Mine in Guizhou Province, China. The Guizhou Province and Liupanshui City maps were downloaded from the National Platform for Common Geospatial Information Services, China / Tianditu, available at: https://cloudcenter.tianditu.gov.cn/administrativeDivision/. The map approval number is GS(2024)0650. The maps were edited by the authors only to indicate the study area, and the administrative boundaries were not modified. **(b)** Simplified stratigraphic column of the roadway area, drawn by the authors based on field geological records from Juxin Coal Mine. **(c)** TBM excavation layout of the #10704 transport roadway and adjacent roadways, independently redrawn by the authors in PowerPoint based on field CAD engineering drawings from Juxin Coal Mine. The field CAD engineering drawings were provided by Juxin Coal Mine and used with permission for academic publication. No copyrighted third-party image was reused.The excavation was carried out using the Liangdu TBM, which has an excavation diameter of 4.5 **m.** As shown in Fig 5, the cutterhead is manufactured from high-strength alloy to withstand the complex mechanical response of the jointed rock mass. The cutterhead is equipped with 30 disc cutters distributed over the excavation face. Each cutter has a nominal diameter of 17 in. (432 mm) and a cutter-tip width of 13 mm, while the center-to-center spacing between adjacent cutters is 89 mm. This cutter layout was selected to suit the highly jointed limestone formation encountered along the roadway and to promote effective crack coalescence and uniform fragment formation during excavation.

As shown in Fig 4, an open TBM was used to excavate the #10704 transport roadway at Juxin Coal Mine, Liupanshui, Guizhou, China. The roadway reached a maximum burial depth of 472 m and followed a straight upward alignment with a gradient of +0.3%, extending from an elevation of 628.9 m to 1095 m over a total length of 2126.8 m. The excavation passed through layered limestone with a uniaxial compressive strength (UCS) of 60–80 MPa, indicating high intact rock strength.

The rock mass was moderately fractured, with an average rock quality designation (RQD) of 70%. Three to four dominant joint sets were developed, significantly influencing the structural integrity and mechanical response of the rock mass. Variations in joint orientation and spacing induced pronounced heterogeneity and anisotropy, which affected TBM performance and excavation stability. The combined influence of joint characteristics and rock strength underscores the necessity of geotechnical evaluation for optimizing TBM operating parameters and maintaining excavation stability. Although the rock mass in the field contains three to four dominant joint sets and exhibits pronounced heterogeneity and anisotropy, the present numerical study adopts a simplified joint configuration. The reason is that this work aims to clarify the dominant failure and fragmentation mechanisms in the local cutter-rock interaction zone, rather than to fully reconstruct the entire structural complexity of the tunnel face. At the cutter scale, the rock-breaking response is mainly governed by several first-order factors, particularly joint inclination, joint spacing, and confining pressure. If multiple joint sets are introduced simultaneously, the interactions among discontinuities become highly coupled, which would obscure the individual contribution of each controlling factor and reduce the interpretability of the parametric analysis. This treatment provides a reasonable basis for mechanistic analysis, while the effects of multi-joint combinations will be considered in future work.

**Fig 5:**
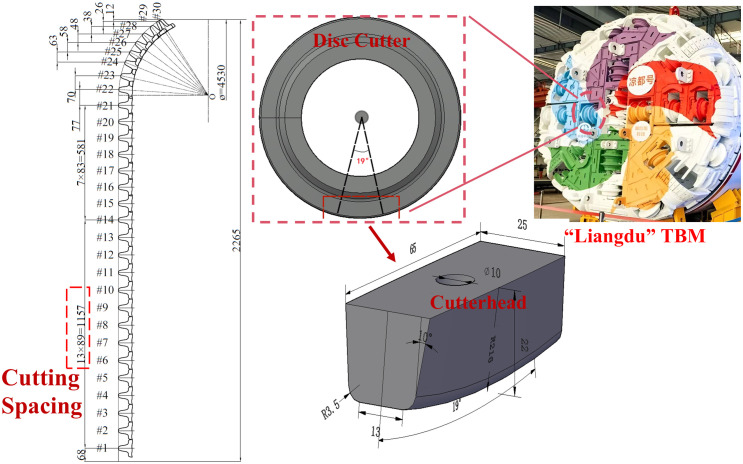
Cutterhead layout of the Liangdu TBM, showing the cutter arrangement, nominal cutter diameter (17 in.), cutter-tip width (13 mm), and adjacent cutter spacing (89 mm).

**Fig 6:**
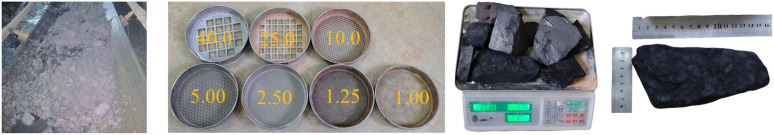
Testing of morphology, size range, and mass of rock fragments.

Rock fragment size analysis was conducted to determine the fragment size distribution. For fragments with relatively large mean sizes, sieve analysis was applied. The sieve set comprised apertures of 40, 25, 10, 5, 2.5, 1.25, and 1.0 mm. The morphology, size distribution, and mass of fragments generated during excavation were recorded (Fig 6).

### 3.2. Model construction

**Fig 7:**
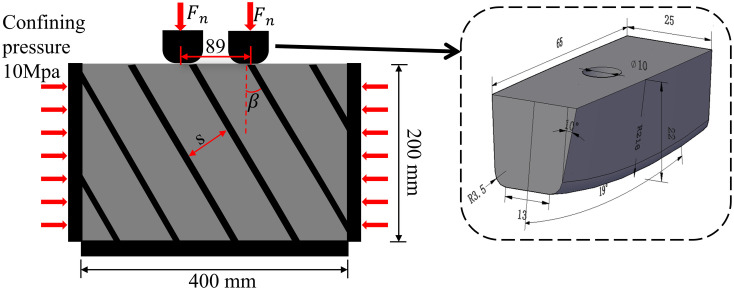
Two-dimensional indentation model for jointed limestone.

To represent the mechanical response of rock under dual-cutter loading, the cutting process was simplified to an indentation problem [34, 40]. A two-dimensional indentation model was established to simulate the response of jointed limestone under cutter loading. The model comprises a limestone specimen 400 mm wide and 200 mm high (Fig 7). Two cutters with constant cross-sections were symmetrically arranged about the specimen centerline with a spacing of 89 mm. Rigid walls were used to impose boundary conditions. The lateral walls applied a confining pressure of 10 MPa to represent in-situ stress, and the bottom wall was fixed to restrict vertical displacement during indentation. These boundary conditions reproduce the mechanical response of the rock under cutter loading.

To quantify crack propagation during indentation, the orientations of bond-break events recorded in DEM were statistically analyzed. In this study, 0° and 180° were defined as horizontal crack propagation, whereas 90° represented vertical propagation. The propagation angle of each crack was determined from the orientation of the line connecting the centers of the two particles at the moment of bond breakage. Tensile and shear cracks were then classified separately and summarized in angular intervals to obtain their directional distributions. The dominant crack propagation angle was defined as the angular interval with the highest crack frequency, which was used to characterize the preferential direction of crack development under different indentation conditions.

The two-dimensional model can be regarded as a plane-strain simplification of the local cutter–rock interaction zone, in which the cutter cross-section is assumed to remain constant along the out-of-plane direction. Although this simplification cannot fully reproduce the three-dimensional contact geometry of an actual disc cutter, it enables controlled comparison of the effects of joint dip angle, joint spacing, and confining pressure on crack propagation, fragment distribution, and energy dissipation. Therefore, the 2D model is suitable for identifying the main fragmentation trends, while its limitation relative to real 3D cutting conditions should be acknowledged.

### 3.3. Macro-parameter determination

Rock specimens were obtained from Juxin Coal Mine for laboratory testing. The specimens were prepared as cylinders 50 mm in diameter and 100 mm in height. The joints were characterized by a dip angle of 60°, a spacing of 10 mm, and a width of 3 mm.

The specimens were installed in a triaxial chamber and subjected to triaxial compression. Confining pressure was applied at 0.5 MPa/min to 10 MPa and maintained for 5 min to achieve stress equilibration. Axial loading was then applied at 0.02 mm/s. Axial force, axial displacement, confining pressure, and lateral deformation were continuously recorded. The tests were terminated at 15% axial strain to characterize the failure behavior of the jointed limestone.

The macroscopic mechanical parameters, including compressive strength, elastic modulus, cohesion, and friction angle, are summarized in Table 1.

**Table 1:** Results of triaxial compression test.

Density (kg/m^3^)	Elastic modulus（GPa）	Compressive strength (MPa)	Cohesion（MPa）	Friction angle （°）
2700	12.64	116.78	53.1	50

### 3.4. Micro-parameter calibration

To ensure that the mesoscopic parameters of the DEM model could adequately reproduce both the compressive and tensile behavior of the jointed limestone, a combined calibration strategy based on the uniaxial compression test and the Brazilian splitting test was adopted. In the simulation, the uniaxial compression specimen measured 100 mm × 50 mm and consisted of 10,298 particles with radii ranging from 0.8 to 1.5 mm. The specimen was enclosed by rigid boundaries, and axial loading was imposed by vertically moving the upper and lower walls under displacement control until an axial strain of 15% was reached. In addition, a Brazilian splitting model was established to reproduce the indirect tensile response of the rock, in which diametral loading was applied through rigid platens.

**Fig 8:**
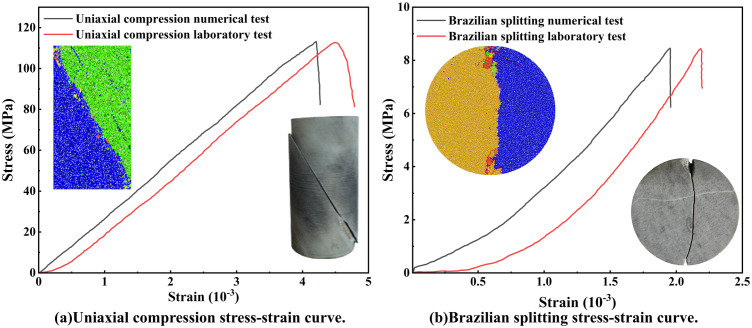
Macro–micro failure mechanism of stress–strain curves.

A trial-and-error calibration procedure was employed to determine the mesoscopic parameters. The particle modulus, stiffness ratio, bond tensile strength, bond cohesion, and inter-particle friction coefficient were progressively adjusted until the numerical results matched the laboratory responses in terms of strength, deformation characteristics, and failure pattern. As shown in Fig 8, the calibrated model reproduced the elastic slope, peak strength, and post-peak failure characteristics obtained from the uniaxial compression test, while also capturing the tensile strength and central splitting failure observed in the Brazilian test. In addition, the simulated crack evolution along the joint plane agreed well with the experimental fracture features. These results indicate that the calibrated parameter set can reasonably characterize the mechanical behavior and fracture process of the jointed rock. The calibrated micro-parameters are summarized in Table 2, and their detailed definitions are given in Table 3.

**Table 2:** Micromechanical parameters of PFC.

Rock type	Micromechanical parameters				
	Rmax/Rmin	$R_{min}(mm)$	$\rho (kg/m^{3})$	φ	kratio
limestone	1.4	0.8	2700	0.07	1.42
joints	1.4	0.8	2500	0.07	2.20
Interface	1.4	0.8	2500	0.07	2.20
	$E^{*}(GPa)$	${\overset{―}{\sigma }}_{c}(MPa)$	$\overset{―}{c}$ ***(MPa)***	$\overset{―}{\phi }(\circ )$	μ
limestone	150	98.20	53.1	50	0.5
joints	75	24.00	11.50	38.325	0.5
interface	75	4.80	2.30	38.325	0.5

**Table 3:** The implication of micro parameters.

$\begin{array}{c} R_{min} \end{array}$	Minimum particle radius	$\begin{array}{c} E^{*} \end{array}$	Effective modulus of the particle
$R_{max}/R_{min}$	The ratio of maximum to minimum particle radius	${\overset{―}{\sigma }}_{c}$	Tensile strength of the parallel bond
ρ	Particle density	$\overset{―}{c}$	The cohesion of the parallel bond
φ	Porosity between particles	$\overset{―}{\phi }$	Friction angle of the parallel bond
kratio	Normal-to-shear stiffness ratio of the particle	μ	Friction coefficient

It should be emphasized, however, that the present calibration was performed under a fixed discretization condition. The particle radius range was set to 0.8–1.5 mm, and the total particle number was kept constant in the calibration model so as to achieve a compromise between computational efficiency and the ability of the bonded-particle model to reproduce the laboratory responses. No dedicated sensitivity analysis was carried out with respect to particle resolution, particle-size distribution, or total particle number. Since DEM predictions can be influenced by the discretization scheme and model scale, these factors may affect not only the simulated macroscopic mechanical response but also the fragment gradation obtained from post-fracture analysis. Therefore, the current parameter set should be regarded as valid for the present discretization level, and further sensitivity analyses are needed in future work to quantify the influence of particle resolution and model size on the robustness of the proposed numerical framework.

### 3.5. Results convergence and validation

**Fig 9:**
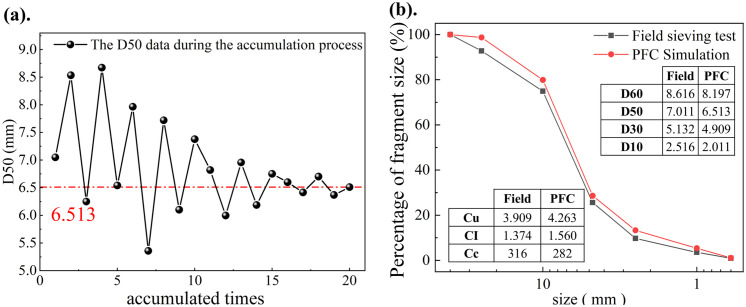
a. Variation of D50 with the accumulated number of simulations; b. Comparison between the Art Auditcumulative average numerical gradation curve after 20 simulations and the field sieving result.

To reduce the stochastic variability in discrete element simulations arising from random particle generation and bond distribution, repeated calculations were performed to obtain stable and representative fragmentation results [41]. In this study, the particle-size distribution curve from each simulation was recorded and cumulatively averaged, and D50 was adopted as the convergence indicator. As shown in Fig 9a, the fluctuation of D50 decreased progressively with increasing simulation number and became essentially stable by the 20th realization [42]. Therefore, 20 repeated simulations were conducted for each working condition in the subsequent analyses.

The cumulative average gradation curve after 20 simulations is presented in Fig 9b. The numerical gradation result agrees well with the field sieving curve, indicating that the adopted model configuration and calibrated parameter set can reasonably reproduce the overall fragmentation characteristics of the excavated rock fragments. These repeated simulations were intended to reduce the stochastic variability inherent in DEM calculations rather than to evaluate the sensitivity of the results to particle resolution or particle-size distribution. Because only a limited number of condition levels were considered for each factor group, the fitted relationships presented in the following sections should be interpreted as first-order trend models under the investigated conditions rather than universal predictive equations.

## 4. Results and discussion

This study examines the effects of joint dip angle, spacing, and confining pressure on crack propagation, with emphasis on failure mechanisms and energy dissipation under varying stress conditions [7, 19, 22]. The objective is to clarify the micro-scale relationship between rock fragment size distribution and TBM cutter efficiency.

### 4.1. Effect of joint dip angles

**Fig 10:**
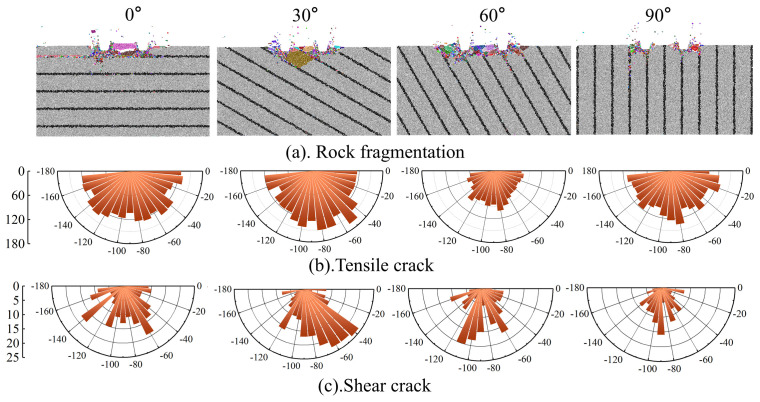
Fragments and crack distributions of the model under different joint dip angles.

The simulated fragmentation pattern varies markedly with joint dip angle, as illustrated in Fig 10a. As the dip angle increased from 0° to 30°, the fragmentation zone under dual-cutter loading remained limited, and the fragment size distribution was relatively uniform. At 30°, fracture propagation along the joint plane was most developed, accompanied by an increased proportion of coarse fragments. This finding agrees with previous studies reporting 30° as favorable for cutterhead rock breaking [22]. When the dip angle increased to 60°, the fragmentation zone reduced, resulting in a narrower size distribution. At 90°, the fragmentation region was minimal, corresponding to the lowest size dispersion.

Figs 10b, c present the quantitative distribution and spatial orientation of tensile and shear cracks at different joint dip angles. Crack orientations were defined as 0° and 180° for horizontal and 90° for vertical, and the vertical axis represents crack count. As the dip angle increased from 0° to 90°, the total crack number first increased and then decreased. Tensile cracks were most developed at 30°, indicating enhanced fragmentation. Shear crack orientations progressively shifted from horizontal to vertical dominance with increasing dip angle. At 30°, the dominant shear crack orientation aligned with the joint plane, indicating pronounced joint-controlled failure.

**Fig 11:**
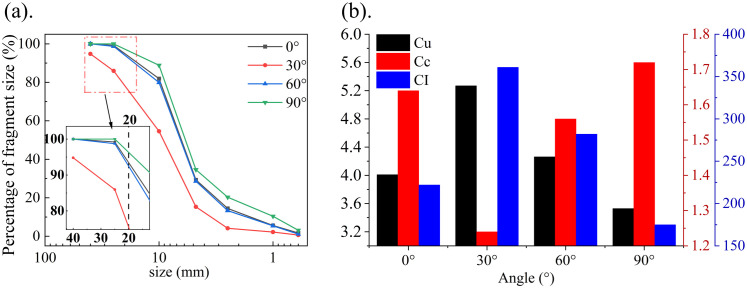
Cumulative average gradation curves and parameters under different joint dip angles.

Fig 11 presents the cumulative gradation curves and associated parameters under varying joint dip angles. The 30° curve is flatter, indicating a broader fragment size range and greater dispersion. In contrast, the curves at 0° and 90° are steeper, reflecting more uniform fragment distributions and higher proportions of fine fragments. The Cc decreased and then increased from 0° to 90°, reaching a minimum of 1.24 at 30° and a maximum of 1.72 at 90°. Conversely, the Cu and CI exhibited the opposite trend, peaking at 5.27 and 361 at 30° and decreasing to 3.53 and 175 at 90°. These findings align with variations in the uniformity index of rock cuttings reported by Song et al. [43].

**Fig 12:**
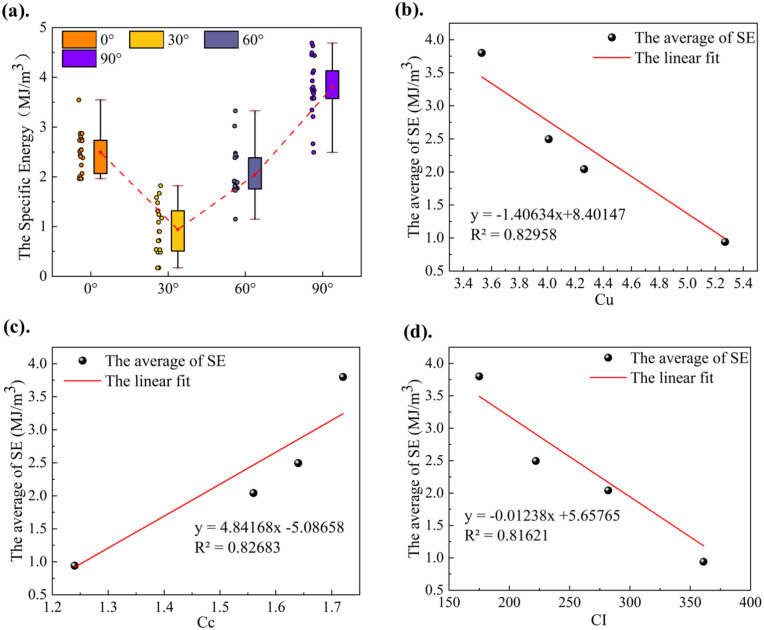
a. SE values under different joint dip angles; b–d. Correlations of Cc, Cu, and CI with SE.

Fig 12a shows the SE under different joint dip angles. A V-shaped trend is observed, with the minimum SE at 30° and the maximum at 90°, indicating that rock breakage is most energy-efficient at an intermediate joint dip angle. This observation is consistent with Kang et al. [44], who reported inclination-dependent energy responses in jointed rock systems. The repeated simulation results also show a certain degree of scatter at each condition, reflecting the inherent stochastic variability of DEM fragmentation. As shown in Fig 12b–d, linear fits between SE and the gradation parameters yield R² values of 0.82, 0.82, and 0.81 for Cc, Cu, and CI, respectively. These relationships suggest that the gradation parameters vary consistently with the overall trend of energy consumption under different joint dip angles. Because only a limited number of dip-angle levels were considered, the fitted relationships should be interpreted as first-order trend models under the investigated conditions rather than universal predictive equations. Nevertheless, the results support the potential of using fragment-size characteristics as practical indicators for evaluating TBM excavation performance under varying joint dip angles.

### 4.2. Effect of confining pressure

**Fig 13:**
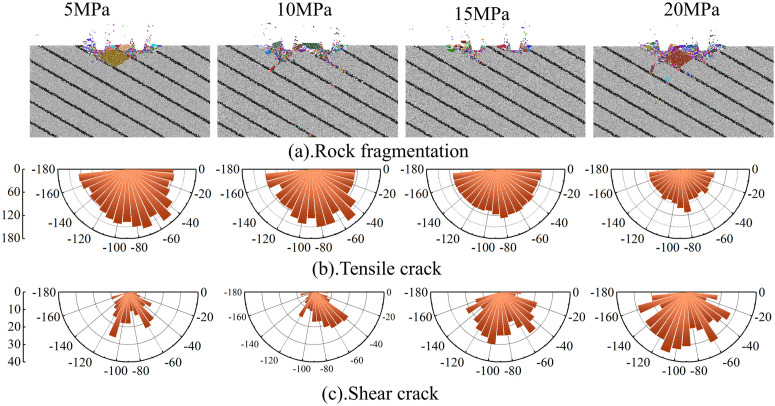
Fragments and crack distributions of the model under different confining pressure.

The influence of confining pressure on fragment distribution is illustrated in Fig 13a. At 5 MPa, fracture along the joint plane is most developed, accompanied by a greater proportion of coarse fragments. This observation agrees with Altindag [18], who reported that low confining pressure produces larger fragments than high confining pressure. As the confining pressure increases to 10 and 15 MPa, the fragmentation zone induced by double-cutter loading decreases, resulting in a narrower size distribution. At 20 MPa, the fragmentation region is minimal, corresponding to the lowest size dispersion. Fig 13b, c present the distribution and orientation of tensile and shear cracks under different confining pressures. As confining pressure increases from 5 to 20 MPa, the number of tensile cracks decreases due to enhanced compressive constraint, whereas shear cracks increase as elevated confinement drives shear stress toward the shear strength. Similar trends were reported by Wu et al. [19].

**Fig 14:**
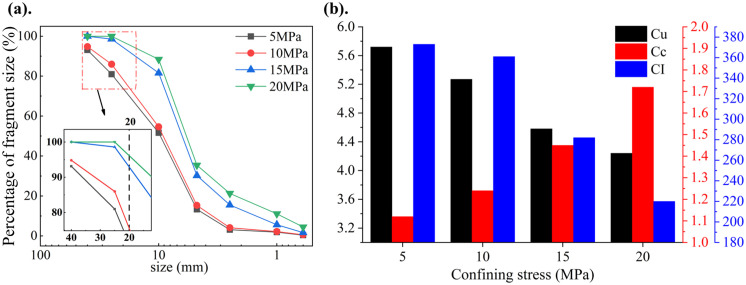
Cumulative average gradation curves and parameters under different confining pressures.

Fig 14a presents the cumulative gradation curves at different confining pressures. At 5 and 10 MPa, the curves are flatter, indicating broader fragment size distributions and greater dispersion. At 15 and 20 MPa, the curves become steeper, reflecting more uniform distributions with higher proportions of fine fragments. As shown in Fig 14b, the Cc increases from 1.12 at 5 MPa to 1.72 at 20 MPa. In contrast, the Cu and CI decrease from 5.72 and 373 at 5 MPa to 4.24 and 220 at 20 MPa. These trends are consistent with Wu et al. [19], who reported that higher confining pressure shifts fragment sizes toward finer fractions.

**Fig 15:**
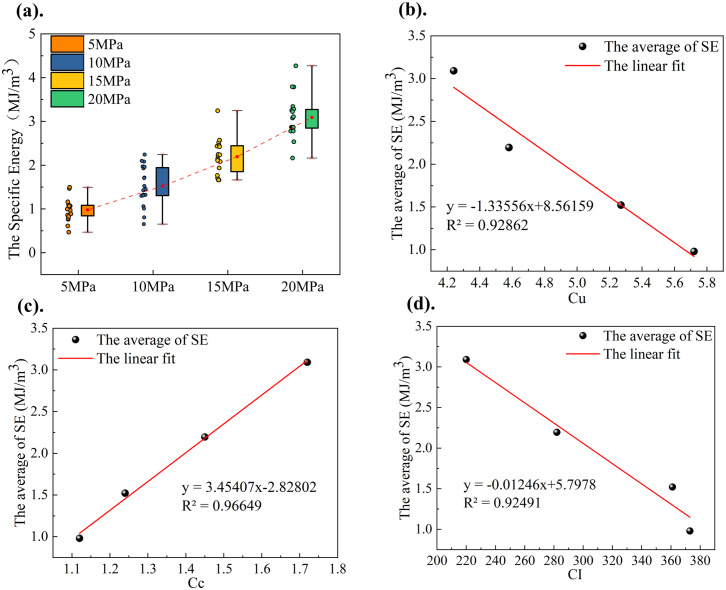
a. SE values under different confining pressures; b–d. Correlations of Cc, Cu, and CI with SE.

Fig 15a shows the SE under different confining pressures. SE increases progressively with confining pressure, reaching a minimum at 5 MPa and a maximum at 20 MPa. This trend is consistent with the suppression of tensile crack propagation and the increased energy demand for rock fracture under higher confinement, in agreement with Ma et al. [23]. The repeated simulation results under each confining-pressure level exhibit limited but visible dispersion, indicating that stochastic variability remains present in the DEM calculations even after cumulative averaging. As shown in Fig 15b–d, the fitted linear relationships between SE and the gradation parameters yield R² values of 0.92, 0.96, and 0.92 for Cc, Cu, and CI, respectively. These relatively high coefficients indicate that the gradation parameters vary consistently with SE over the investigated confining-pressure range. However, because the number of confining-pressure levels considered in this study is still limited, these fitted relationships are more appropriately interpreted as first-order trend models within the present test range. Within this scope, the results support the use of fragment gradation as a quantitative indicator of TBM rock-breaking efficiency under different confining-pressure conditions.

### 4.3. Effect of joint spacing

**Fig 16:**
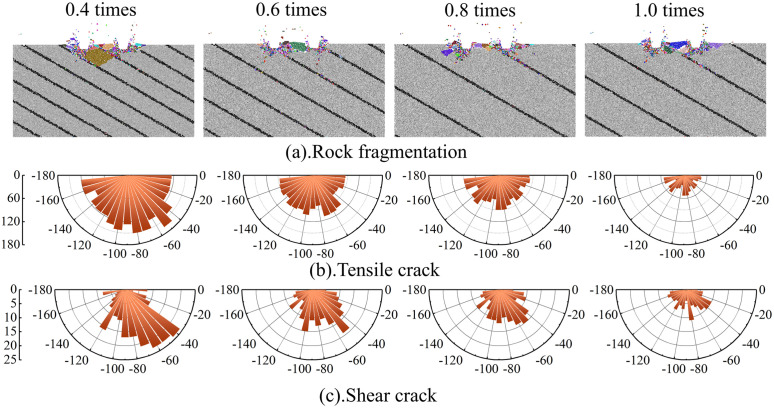
Fragment and crack distributions of the model under different joint spacings.

To evaluate the influence of joint spacing on dual-cutter efficiency, joint spacing was normalized by the cutter spacing (89 mm). As shown in Fig 16a, varying joint spacings result in distinct fragmentation patterns, while Fig 16b, c present the corresponding crack propagation characteristics.

At a spacing of 0.4 times the cutter spacing, dense joints markedly enhance the initiation and propagation of tensile and shear cracks. Tensile cracks are widely distributed with large propagation extent, whereas shear cracks form interconnected networks. These conditions promote rapid crack coalescence and effective fragmentation along joint planes, consistent with Bejari and Khademi Hamidi [7]. As joint spacing increases, fragmentation intensity decreases and the affected zone contracts. The angular range and extent of tensile cracks are reduced, and shear cracks become more localized, weakening crack interaction and network formation. At 1.0 times the cutter spacing, fragmentation is limited and localized, producing only a few coarse fragments. Crack development is suppressed, and deformation shifts toward plastic yielding rather than effective fracturing.

**Fig 17:**
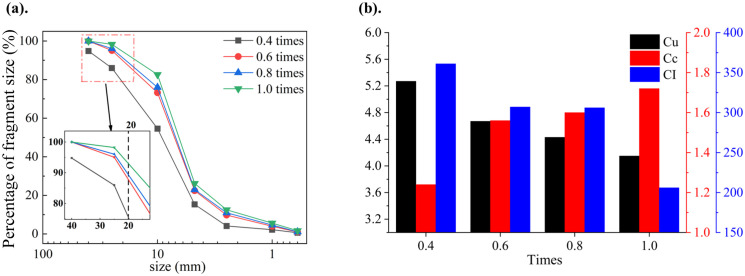
Cumulative average gradation curves and parameters under different joint spacings.

Fig 17a presents the cumulative gradation curves for different joint spacings. As spacing increases from 0.4 to 1.0 times the cutter spacing, fragments larger than 20 mm dominate at 0.4 times, whereas finer fragments prevail at 1.0 times, consistent with Shaterpour-Mamaghani and Bilgin [11]. Fig 17b shows the variation of Cu, Cc, and CI with joint spacing. The Cc increases from 1.24 at 0.4 times to 1.73 at 1.0 times, whereas Cu and CI decrease from 5.27 and 361 to 4.15 and 206, respectively.

**Fig 18:**
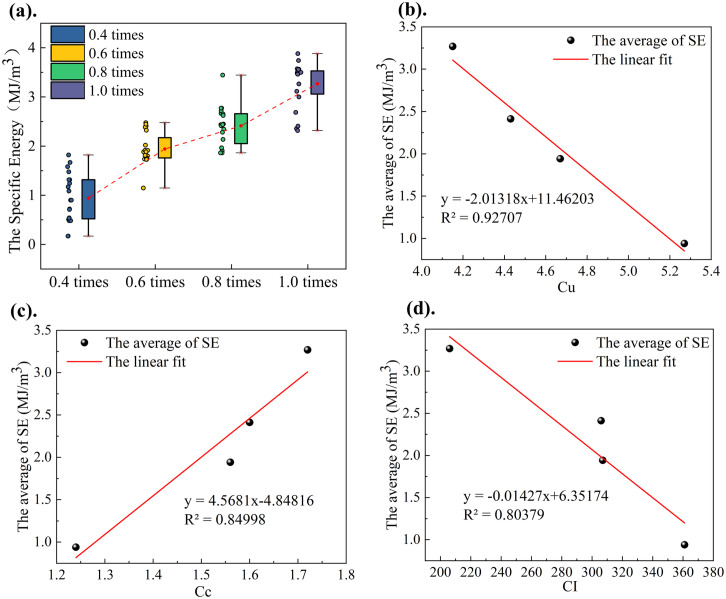
a. SE values under different joint spacings; b–d. Correlations of Cc, Cu, and CI with SE.

The variation in SE with joint spacing is shown in Fig 18a. SE is lowest at 0.4 times the cutter spacing and increases gradually with spacing, indicating that denser joints promote more efficient crack interaction and lower energy consumption during rock breakage. This trend is consistent with the numerical observations reported by Xue et al. [45]. As in the other cases, the repeated simulation results show some dispersion at each spacing level, which reflects the inherent heterogeneity of DEM fragmentation. The fitted relationships shown in Fig 18b–d yield R² values of 0.92, 0.84, and 0.80 for Cc, Cu, and CI, respectively. These results indicate that the variation of gradation parameters is broadly consistent with the variation in SE under different joint spacings. Because only four spacing levels were considered, the fitted linear relationships should be interpreted as first-order trend descriptions for the present numerical conditions rather than universal predictive models. Even so, the observed correlations provide useful support for the practical use of fragment gradation in assessing TBM excavation performance under varying joint-spacing conditions.

## 5. Potential application

In karst-dominated coal mines, roadway excavation is carried out under complex geological conditions characterized by high rock strength, pronounced heterogeneity, and extensive jointing [26]. These factors increase the difficulty of TBM rock breaking and the associated operational risks [6]. Inappropriate operating parameters may reduce excavation efficiency, accelerate cutter wear, and increase the risk of machine jamming and gas outbursts [27]. Therefore, reliable real-time indicators are needed to support safe and efficient TBM operation.

The numerical results reveal clear correlations between rock-breaking efficiency and gradation parameters (Cu, Cc, and CI). This suggests that fragment size distribution can reflect cutter–rock interaction behavior and excavation efficiency under complex geological conditions. Accordingly, gradation parameters may serve as practical indicators for evaluating TBM performance in karst coal mine roadways. With automated fragment identification systems, these parameters can be acquired in real time using image recognition and segmentation techniques [10, 25].

**Fig 19:**
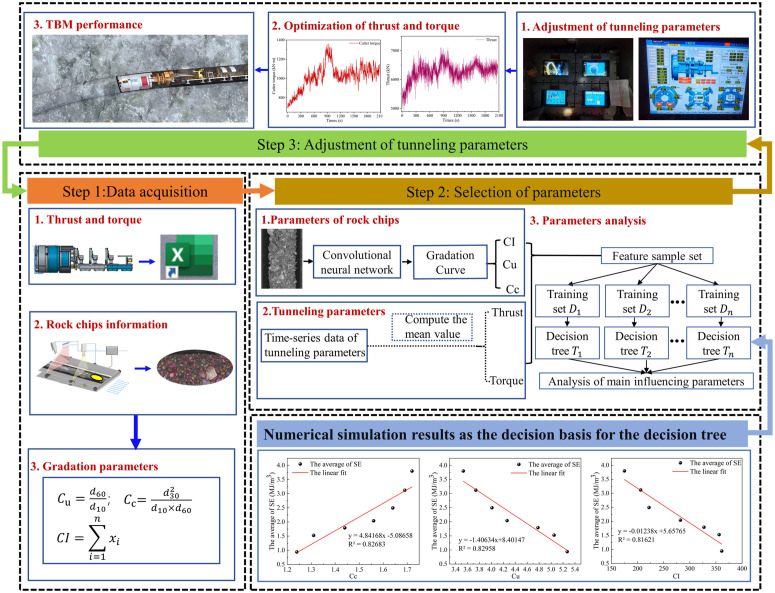
Field application process.

As shown in Fig 19, a data-driven framework for real-time TBM performance optimization is proposed. The framework integrates multi-source data, including TBM operational parameters and rock fragment characteristics. Fragment images are processed to extract key gradation parameters, which are then combined with operational data to support the adjustment of tunneling parameters such as thrust and torque. Under the investigated conditions, this framework provides a potential basis for adaptive TBM control in strongly jointed and high-stress strata.

## 6. Conclusion

This study examined the relationship between rock fragment gradation and TBM cutter rock-breaking efficiency in jointed rock masses using discrete element simulations of double-cutter indentation. The influences of joint dip angle, joint spacing, and confining pressure were systematically evaluated, and the correlations between gradation parameters and SE were quantified. The main findings are summarized as follows:

1. The strong correlations between gradation parameters (Cu, Cc, CI) and SE suggest that fragment size distribution quantitatively characterizes cutter–rock interaction intensity and energy consumption during rock breakage.
2. Joint dip angle and spacing significantly influence tensile–shear crack initiation, propagation, and coalescence.
3. Increasing confining pressure suppresses tensile cracking and promotes shear-dominated or plastic deformation, resulting in finer fragments and higher SE.
4. The statistical relationships between Cu, Cc, CI, and SE establish a quantitative link between fragment characteristics and excavation efficiency, enabling indirect assessment of cutter performance in complex geological conditions.

The results indicate that rock fragment gradation can serve as a quantitative indicator for real-time assessment of TBM excavation efficiency. The proposed framework provides a basis for optimizing tunneling parameters in karst coal mines and other jointed strata. However, the effects of fragment disturbance during field handling and transportation were not considered and may influence fragment morphology. Future work will incorporate machine learning and automated fragment recognition to improve the accuracy and robustness of TBM performance evaluation and control.

## Supporting information

S1 File All original DEM simulation codes used in this study. (DOCX)
