# Supplementary material for: Quantitative correlation of rock fragment gradation parameters and TBM disc cutter efficiency: Insights from DEM simulations
Source: PLoS One. 2026 Jun 26;21(6):e0345490. doi: 10.1371/journal.pone.0345490 (PMC13308788; doi:10.1371/journal.pone.0345490)
Supplement: S1 File — (DOCX) [file pone.0345490.s001.docx]

**S1:**

program call '1chengyang'

program call '2yuya'

program call '3jiajiaojie'

program call '4yinglishifang'

program call '5jiagundao'

program call '6jiazai'

program call '7fenzu'

**S2:**

model new

def par

width=0.3

height=0.2

rdmin=0.0008

rdmax=rdmin*1.4

poro=0.07

end

@par

model domain extent [-width*2.0] [width*2.0] ...

[-height*2.0] [height*2.0]

;model random 10001

wall generate box [-width*0.5] [width*0.5] ...

[-height*0.5] [height*0.5] expand 1.5

ball distribute porosity @poro radius [rdmin] [rdmax] box ...

[-width*0.5] [width*0.5] [-height*0.5] [height*0.5]

ball attribute density 2.7e3 damp 0.7

cmat default model linear method deform emod 100e6 kratio 1.5

model cycle 2000 calm 50

contact property fric 0.5

model solve

model save 'sample'

**S3:**

model restore 'sample'

[txx=-1e5]

[tyy=-1e5]

[sevro_factor=0.2]

[do_xSevro=true]

[do_ySevro=true]

[sevro_freq=100]

[timestepNow=global.step-1]

def sevro_walls

compute_stress

if timestepNow<global.step then

get_g(sevro_factor)

timestepNow+=sevro_freq

endif

if do_xSevro=true then

Xvel=gx*(wxss-txx)

wall.vel.x(wpRight)=-Xvel

wall.vel.x(wpLeft)=Xvel

endif

if do_ySevro=true then

Yvel=gy*(wyss-tyy)

wall.vel.y(wpUp)=-Yvel

wall.vel.y(wpDown)=Yvel

endif

end

def wp_ini

wpDown=wall.find(1)

wpRight=wall.find(2)

wpUp=wall.find(3)

wpLeft=wall.find(4)

end

@wp_ini

def computer_chiCun

wlx=wall.pos.x(wpRight)-wall.pos.x(wpLeft)

wly=wall.pos.y(wpUp)-wall.pos.y(wpDown)

end

def compute_stress

computer_chiCun

wxss=-(wall.force.contact.x(wpRight)-wall.force.contact.x(wpLeft))*0.5/wly

wyss=-(wall.force.contact.y(wpUp)-wall.force.contact.y(wpDown))*0.5/wlx

end

@compute_stress

def get_g(fac)

computer_chiCun

gx=0

gy=0

zongKNX=100e6*10

zongKNY=100e6*10

loop foreach ct wall.contactmap(wpLeft)

zongKNX+=contact.prop(ct,"kn")

endloop

loop foreach ct wall.contactmap(wpRight)

zongKNX+=contact.prop(ct,"kn")

endloop

loop foreach ct wall.contactmap(wpUp)

zongKNY+=contact.prop(ct,"kn")

endloop

loop foreach ct wall.contactmap(wpDown)

zongKNY+=contact.prop(ct,"kn")

endloop

gx=fac*wly/(zongKNX*global.timestep)

gy=fac*wlx/(zongKNY*global.timestep)

end

@compute_stress

fish callback add @sevro_walls -1.0

history id 1 @wxss

history id 2 @wyss

model cycle 1

model solve

model save 'yuya'

**S4:**

model restore 'yuya'

[jianju=0.05]

[kuandu=0.005]

[jiaodu=-45]

ball group 'yanshi'

ball group 'cengli' range plane origin 0 0 dip [jiaodu] distance [kuandu*0.5]

def add_ruan_up

pos_x=0

pos_y=0

loop while pos_x<wlx*0.5

pos_x+=(jianju+kuandu)*math.cos(jiaodu*math.pi/180.0)

pos_y+=(jianju+kuandu)*math.sin(jiaodu*math.pi/180.0)

command

ball group 'cengli' range plane origin [pos_x] [pos_y] dip [jiaodu] distance [kuandu*0.5]

endcommand

endloop

end

@add_ruan_up

def add_ruan_down

pos_x=0

pos_y=0

loop while pos_x>-wlx*0.5

pos_x-=(jianju+kuandu)*math.cos(jiaodu*math.pi/180.0)

pos_y-=(jianju+kuandu)*math.sin(jiaodu*math.pi/180.0)

command

ball group 'cengli' range plane origin [pos_x] [pos_y] dip [jiaodu] distance [kuandu*0.5]

endcommand

endloop

end

@add_ruan_down

[ying_pb_coh=53.1e7]

[ying_pb_ten=ying_pb_coh*0.7]

[ruan_pb_coh=ying_pb_coh*0.22]

[ruan_pb_ten=ruan_pb_coh*0.7]

[fai=50]

[fai2=fai*0.7]

[emod=150e9]

[emod2=emod*0.43]

cmat add 1 model linear method deform emod 1e9 kratio 1.5 range contact type 'ball-facet'

cmat add 2 model linearpbond method deform emod [emod] kratio 1.5 ...

pb_deform emod [emod] kratio 1.5 ...

property pb_coh [ying_pb_coh] pb_ten [ying_pb_ten] pb_fa [fai] fric 0.5 ...

range group 'yanshi'

cmat add 3 model linearpbond method deform emod [emod2] kratio 1.5 ...

pb_deform emod [emod2] kratio 1.5 ...

property pb_coh [ruan_pb_coh] pb_ten [ruan_pb_ten] pb_fa [fai2] fric 0.5 ...

range group 'cengli'

contact cmat apply

model clean

model cycle 1

model solve

model save 'temp1'

contact method bond gap [rdmin*0.2]

model cycle 1

model solve

model save 'jiajiaojie'

**S5:**

model restore 'jiajiaojie'

wall attribute vel 0

[txx=-10e6]

[tyy=-10e6]

def computer_chicun

wlx=wall.pos.x(wpright)-wall.pos.x(wpleft)

end

def computer_stress

computer_chicun

wxss=0.5*(wall.force.contact.x(wpleft)-wall.force.contact.x(wpright))/wly

end

def get_g

zongKNX=2e10*2.0

loop foreach ct wall.contactmap(wpleft)

zongKNX+=contact.prop(ct,"kn")

endloop

loop foreach ct wall.contactmap(wpright)

zongKNX+=contact.prop(ct,"kn")

endloop

gx=1.0*servo_factor*wly/(zongKNX*global.timestep)

end

def sevro_walls

computer_stress

if global.step>time_record then

get_g

time_record=global.step+sevro_freq

endif

xvel=gx*math.abs(math.abs(wxss)-txx)

end

wall delete walls range id 3

model cycle 1

model solve

model save 'init_state'

**S6:**

model restore 'init_state'

def daopianxingzhuang

downwide=width*0.04

downHeight=downwide*7

height=downHeight*0.6

upwide=downwide+math.tan(10*math.pi/180.0)*downHeight*0.8

kuadu=0.089

end

@daopianxingzhuang

def creatDaopianZuo

zhongxinX=-0.5*kuadu

zhongxinY=0.5*wly

command

wall create vertices [zhongxinX+0.5*downwide] [zhongxinY] [zhongxinX+0.6*downwide] [zhongxinY+downHeight*0.06] ...

[zhongxinX+0.6*downwide] [zhongxinY+downHeight*0.06] [zhongxinX+0.5*upwide] [zhongxinY+downHeight*0.3] ...

[zhongxinX+0.5*upwide] [zhongxinY+downHeight*0.3] [zhongxinX+0.5*upwide] [zhongxinY+height*0.6] ...

[zhongxinX+0.5*upwide] [zhongxinY+height*0.6] [zhongxinX-0.5*upwide] [zhongxinY+height*0.6] ...

[zhongxinX-0.5*upwide] [zhongxinY+height*0.6] [zhongxinX-0.5*upwide] [zhongxinY+downHeight*0.3] ...

[zhongxinX-0.5*upwide] [zhongxinY+downHeight*0.3] [zhongxinX-0.6*downwide] [zhongxinY+downHeight*0.06] ...

[zhongxinX-0.6*downwide] [zhongxinY+downHeight*0.06] [zhongxinX-0.5*downwide] [zhongxinY] ...

[zhongxinX-0.5*downwide] [zhongxinY] [zhongxinX+0.5*downwide] [zhongxinY] id 5

endcommand

end

def creatDaopianYou

zhongxinX=0.5*kuadu

zhongxinY=0.5*wly

command

wall create vertices [zhongxinX+0.5*downwide] [zhongxinY] [zhongxinX+0.6*downwide] [zhongxinY+downHeight*0.06] ...

[zhongxinX+0.6*downwide] [zhongxinY+downHeight*0.06] [zhongxinX+0.5*upwide] [zhongxinY+downHeight*0.3] ...

[zhongxinX+0.5*upwide] [zhongxinY+downHeight*0.3] [zhongxinX+0.5*upwide] [zhongxinY+height*0.6] ...

[zhongxinX+0.5*upwide] [zhongxinY+height*0.6] [zhongxinX-0.5*upwide] [zhongxinY+height*0.6] ...

[zhongxinX-0.5*upwide] [zhongxinY+height*0.6] [zhongxinX-0.5*upwide] [zhongxinY+downHeight*0.3] ...

[zhongxinX-0.5*upwide] [zhongxinY+downHeight*0.3] [zhongxinX-0.6*downwide] [zhongxinY+downHeight*0.06] ...

[zhongxinX-0.6*downwide] [zhongxinY+downHeight*0.06] [zhongxinX-0.5*downwide] [zhongxinY] ...

[zhongxinX-0.5*downwide] [zhongxinY] [zhongxinX+0.5*downwide] [zhongxinY] id 6

endcommand

end

@creatDaopianZuo

@creatDaopianYou

model save 'jiadaopian'

**S7:**

model restore 'jiadaopian'

model orientation-tracking on

[daopian_vel=5]

[shendu=0.01]

model mechanical time-total 0

ball attribute displacement multiply 0

wall attribute vel-y [-daopian_vel] range id 5 6

program call 'fracture.p2fis'

@track_init

[wp_daopian_1=wall.find(5)]

[wp_daopian_2=wall.find(6)]

def jiance

whilestepping

faxiangli_5=wall.force.contact.y(wp_daopian_1)

weiyi_1=wall.disp.y(wp_daopian_1)

time= mech.time.total

faxiangli_6=wall.force.contact.y(wp_daopian_2)

weiyi_2=wall.disp.y(wp_daopian_2)

end

history delete

history id 1 @faxiangli_5

history id 2 @faxiangli_6

history id 3 @weiyi_2

history id 4 @weiyi_1

history id 5 @time

history id 6 @wxss

model solve time [shendu/daopian_vel]

model save 'result'

**S8:**

model restore 'result'

define classfied_group

local max_N=0

loop foreach local ball ball.list

local frag = ball.fragment(ball)

ball.extra(ball,3) = frag

if frag>=max_N

max_N=max_N+1

endif

end_loop

loop foreach ball ball.list

local extra_val = ball.extra(ball,3)

if extra_val=0

ball.extra(ball,3)=max_N

max_N=max_N+1

endif

end_loop

loop foreach ball ball.list

ball.group(ball,2)=string(ball.extra(ball,3) )

end_loop

end

@classfied_group

**S9:**

define cal_vol

loop foreach local ball ball.list

table(1,ball.extra(ball,3))= table(1,ball.extra(ball,3))+(ball.vol(ball)*4./3./3.1415926575)^(2./3.)*3.1415926575

end_loop

end

@cal_vol

table 1 export "cc.tab"

; fname: fracture.p2fis

;

; Simple environment to track fragmentation in a BPM.

; Track LinearPBond model "bond_change" events and turn them into fractures.

; Use Fragment logic and Ball Result logic to record fragemnt ids

;

;=============================================================================

fish define add_crack(entries)

local contact = entries(1)

local mode = entries(2)

local frac_pos = contact.pos(contact)

local norm = contact.normal(contact)

local dfn_label = 'crack'

local frac_size

local bp1 = contact.end1(contact)

local bp2 = contact.end2(contact)

local ret = math.min(ball.radius(bp1),ball.radius(bp2))

;contact.method(contact,'pb_radius')

frac_size = ret

local inDir = vector(-comp.y(norm),comp.x(norm))

local vert1 = frac_pos + inDir * frac_size

local vert2 = frac_pos - inDir * frac_size

local arg = array.create(4)

arg(1) = 'vertices'

arg(2) = 2

arg(3) = vert1

arg(4) = vert2

crack_num = crack_num + 1

if mode = 1 then

; failed in tension

dfn_label = dfn_label + '_tension'

else if mode = 2 then

; failed in shear

dfn_label = dfn_label + '_shear'

endif

global dfn = dfn.find(dfn_label)

if dfn = null then

dfn = dfn.create(dfn_label)

endif

local fnew = fracture.create(dfn,arg)

fracture.prop(fnew,'age') = mech.time.total

fracture.extra(fnew,1) = bp1

fracture.extra(fnew,2) = bp2

crack_accum += 1

if crack_accum > 50

if frag_time < mech.time.total

frag_time = mech.time.total

crack_accum = 0

command

fragment compute

endcommand

; go through and update the fracture positions

loop for (local i = 0, i < 2, i = i + 1)

local name = 'crack_tension'

if i = 1

name = 'crack_shear'

endif

dfn = dfn.find(name)

if dfn # null

loop foreach local frac dfn.fracturelist(dfn)

local ball1 = fracture.extra(frac,1)

local ball2 = fracture.extra(frac,2)

if ball1 # null

if ball2 # null

local len=fracture.len(frac)/2.0

local pos=(ball.pos(ball1)+ball.pos(ball2))/2.0

if comp.x(pos)-len > xmin

if comp.x(pos)+len < xmax

if comp.y(pos)-len > ymin

if comp.y(pos)+len < ymax

fracture.pos(frac) = pos

endif

endif

endif

endif

endif

endif

endloop

endif

endloop

endif

endif

end

fish define track_init

command

fracture delete

fragment clear

fragment register ball-ball

endcommand

; activate fishcalls

command

fish callback remove @add_crack

fish callback add @add_crack event bond_break

endcommand

; reset global variables

global crack_accum = 0

global crack_num = 0

global track_time0 = mech.time.total

global frag_time = mech.time.total

global xmin = domain.min.x()

global ymin = domain.min.y()

global xmax = domain.max.x()

global ymax = domain.max.y()

end

;=============================================================================

; eof: fracture.p3fis
